# Solvent-assisted thermal reduction of microcrystalline graphene oxide with excellent microwave absorption performance

**DOI:** 10.1039/c8ra01764f

**Published:** 2018-04-24

**Authors:** Chenbo Liao, Xukun Zhu, Wei Xie, Fangmei Zeng, Shihe Yi, Haifeng Cheng, Jiacai Kuang, Yingjun Deng, Taishan Cao

**Affiliations:** Key Laboratory of Lightweight and Reliability Technology for Engineering Vehicle, The Education Department of Hunan Province, Changsha University of Science & Technology Changsha 410114 China xwxw00@163.com; College of Aerospace Science and Engineering, National University of Defense Technology Changsha 410073 China

## Abstract

To prepare a new kind of electromagnetic wave absorber, and improve the processing technology and accessional value of natural microcrystalline graphite minerals (NMGMs), reduced microcrystalline graphene oxide (rGO-M), a novel absorber with high absorption, low reflection and a wide absorption band, was prepared from NMGMs using a solvent-assisted thermal reduction method. Moreover, the as-produced rGO-M with adjustable electrical resistivity can be easily transferred into well distributed bulk materials by freeze-drying technology. These unique structures and compositions make a great contribution to the impedance match, and cause strong conductive loss and various dipole polarization effects which greatly enhance the absorption. Meanwhile, the effective bandwidths below −5 dB and −10 dB are 11.7 GHz and 3.32 GHz respectively, and the reflection loss can reach −42.68 dB. The study will be beneficial to the development of carbon resources and carbon materials research. Besides, it can provide a scientific basis for the further improvement of the comprehensive utilization and the level of deep processing technology of NMGM resources.

## Abstract 2

Reduced microcrystalline graphene oxide (rGO-M), a novel absorber with high absorption, low reflection and a wide absorption band, was prepared from NMGMs using a solvent-assisted thermal reduction method.

## Graphical abstract

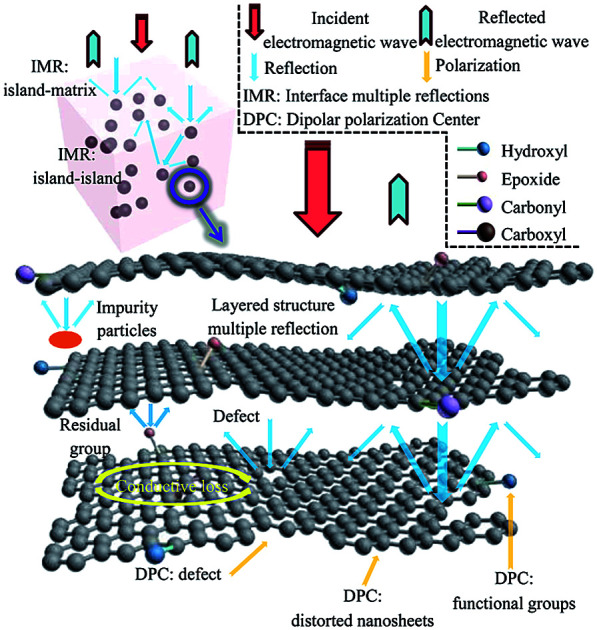

## 1. Introduction

Advanced absorbent composites with various potential applications, such as in aircraft, communication devices, and cutting-edge electronics, are attracting growing attention based on their unique nature.^1–5^ Through different electromagnetic wave dissipation mechanisms, the absorbing-wave materials can be divided into two categories, namely, dielectric and magnetic materials.^6^ In previous applications, ferromagnetic metals and their oxides^7–9^ are often used as absorbents in this field. Although these absorbers operate *via* dielectric and magnetic mechanisms of energy absorption as well as having advantages of low cost and mass production, their disadvantages of density and thermal stability make them lose their development potential when compared to the competition. Conventionally, magnetic materials have better electromagnetic wave absorption properties, but it is unavoidable that the absorption performance is governed by Snoek’s limiting high weight penalty, particularly for applications.^10^ At the same time, carbon-based dielectric absorbers have the disadvantage of narrow bandwidth, but their performance can be improved by constructing different space structures of carbon atoms. In addition, the matching ability between the material and the incident electromagnetic wave (called impedance matching) is also an important index to measure the performance of absorbing materials, which is related to whether the electromagnetic wave can penetrate the material and be absorbed.

Carbon-based materials have been considered as the best choice for electromagnetic shielding and attenuation since World War II. Up until now, a range of carbon materials with different forms, both traditional carbon materials and new nano-carbon materials such as carbon nanotubes,^11–13^ carbon nanofibers,^14–16^ carbon spheres,^17–19^ carbon foams,^13,20^ graphite,^21–23^*etc.*, have been utilized as the main component of novel electromagnetic absorbers. Since it was successfully prepared, graphene has received great attention due to its excellent electrical and thermal conductivity, as well as its superior mechanical properties.^24–26^ As a kind of two-dimensional material, in addition to fullerenes (zero-dimensional), carbon nanotubes (one-dimensional) and graphite (three-dimensional), graphene has supplemented its use and perfected the field of carbon materials. After monolayer graphene was successfully prepared by the micro-mechanical peeling method,^27^ a variety of preparation methods have been reported, such as the oxidation–reduction method,^28–30^ chemical vapor deposition,^31–33^ and chemical synthesis.^34–36^ In particular, the oxidation–reduction method can efficiently prepare graphene under relatively mild conditions, and the defects and residual oxygen functional groups on the reduction products can improve the activity of the reduced graphene oxide (rGO), which is beneficial for expanding its application range. Therefore, as a new kind of nano-filler, graphene has been widely applied to prepare composites.

With the development of graphene and graphene-like materials, the exploration of graphene as an electromagnetic wave absorber has undergone great breakthroughs. However, there are still some problems to be solved, like the inability to realize batch production, agglomeration of graphene and so on. The core problem is that graphene is a non-magnetic material with excellent conductivity, and so the impedance matching characteristics of the material are very poor and the incident electromagnetic wave cannot smoothly penetrate the material, but will be reflected back.^37^ Therefore, finding out how to adjust the conductivity of graphene to control the impedance matching characteristics of materials is an important problem in current research.

The core of our work is to adjust the conductivity of graphene absorbers from many aspects, so as to improve the impedance matching performance of the materials. Herein, NMGMs were chosen as the raw material, and the conductivity of rGO-M was adjusted by the solvent-assisted thermal reduction method. The purpose is to prepare a kind of high quality absorbent.

## 2. Experimental

### 2.1 Preparation process of rGO-M

All of the chemicals in our experiments were analytical grade reagents and were used directly without further treatment. Graphene oxide from natural microcrystalline graphite (GO-M) was prepared by the oxidation of NMGM powder.^38^ The microcrystalline graphene in this paper refers to the graphene prepared from NMGM. The following outlines the preparation of reduced graphene oxides from natural microcrystalline graphite (rGO-M): firstly, GO-M was prepared through the modified Hummers method from natural microcrystalline graphite (obtained from Chenzhou, Hunan province, China). Subsequently, a 120 mg amount of GO-M was dispersed in 40 ml of aqueous solvent by sonication for 0.5 h. Thereafter, the GO-M suspension was treated using the solvothermal method in a Teflon-lined autoclave at 180 °C for 12 hours to form rGO-M intermediate products. Finally, the rGO-M intermediate products were frozen at −50 °C for 6 hours, followed by 24 hours of sublimation under a vacuum of −50 μbar to obtain the rGO-M.

### 2.2 Characterization

The morphology of the samples was identified by field emission scanning electron microscopy (SEM, TESCAN MIRA 3LMU, TescanOrsay Holding, Czech Republic), transmission electron microscopy (TEM, JEM-2100F, Japan Electronics Co. Ltd, Japan) and atomic force microscopy (AFM, PicoPlus, Molecular Imaging, USA). The crystal structure of the samples was identified using an X-ray diffractometer (XRD, D8-Advance, Bruker Corporation, Germany), using Cu target Kα radiation (*λ* = 1.54 Å) with 40 kV scanning voltage, 40 mA scanning current and scanning range from 3° to 70°. In addition, the Raman spectrum of the samples was investigated on a LabRAM HR800 spectrometer (Raman, Horiba Jobin Yvon, France) using laser excitation at 514.5 nm. Moreover, Fourier transform infrared spectroscopy-attenuated total reflectance spectra were recorded on a WQF-510A spectrometer (FT-IR, Beijing Beifen-Ruili Analytical Instrument Co. Ltd, China). The scanning electron microscope is equipped with an Oxford X-Max20 Energy spectrum system for energy dispersive X-ray spectroscopy analysis (EDS). X-ray photoelectron spectra were measured on an X-ray photoelectron spectrometer (XPS, K-alpha 1063, Thermo Scientific, USA) using Al Kα (*hν* 1486.6 eV) X-rays as the excitation source. The electrical resistivity of the samples was measured by a four-probe resistivity meter (RTS-8, Four-probe TECH, China). The most important permittivity (*ε*′ and *ε*′′) and permeability (*μ*′ and *μ*′′) values of the samples with dimensions of concentric rings (inner diameter is 3.04 mm, outer diameter is 7 mm, thickness is 2 to 4 mm, with paraffin matrix of 15 wt%) were measured by a vector network analyzer (Agilent 8720ET, Agilent Technologies Co. Ltd, USA) with the waveguide method at 2–18 GHz.

## 3. Results and discussion

It has been proved that the existence of these defects and residual groups of rGO is beneficial for reducing the conductivity of graphene. In other words, the defects mentioned above are an advantage for graphene-based absorbers and the core problem also offers the chance for a solution. It also provides a possibility for mass production. Some researchers have pointed out that by controlling the purity of graphene and its electronic structure, the conductivity problem of graphene can be solved and the impedance matching problem can be improved. Herein, a straightforward strategy is demonstrated to synthesize reduced microcrystalline graphene oxide with excellent and adjustable electromagnetic absorption performance by a solvent-assisted thermal reduction method starting from NMGM.

**Fig. 1:**
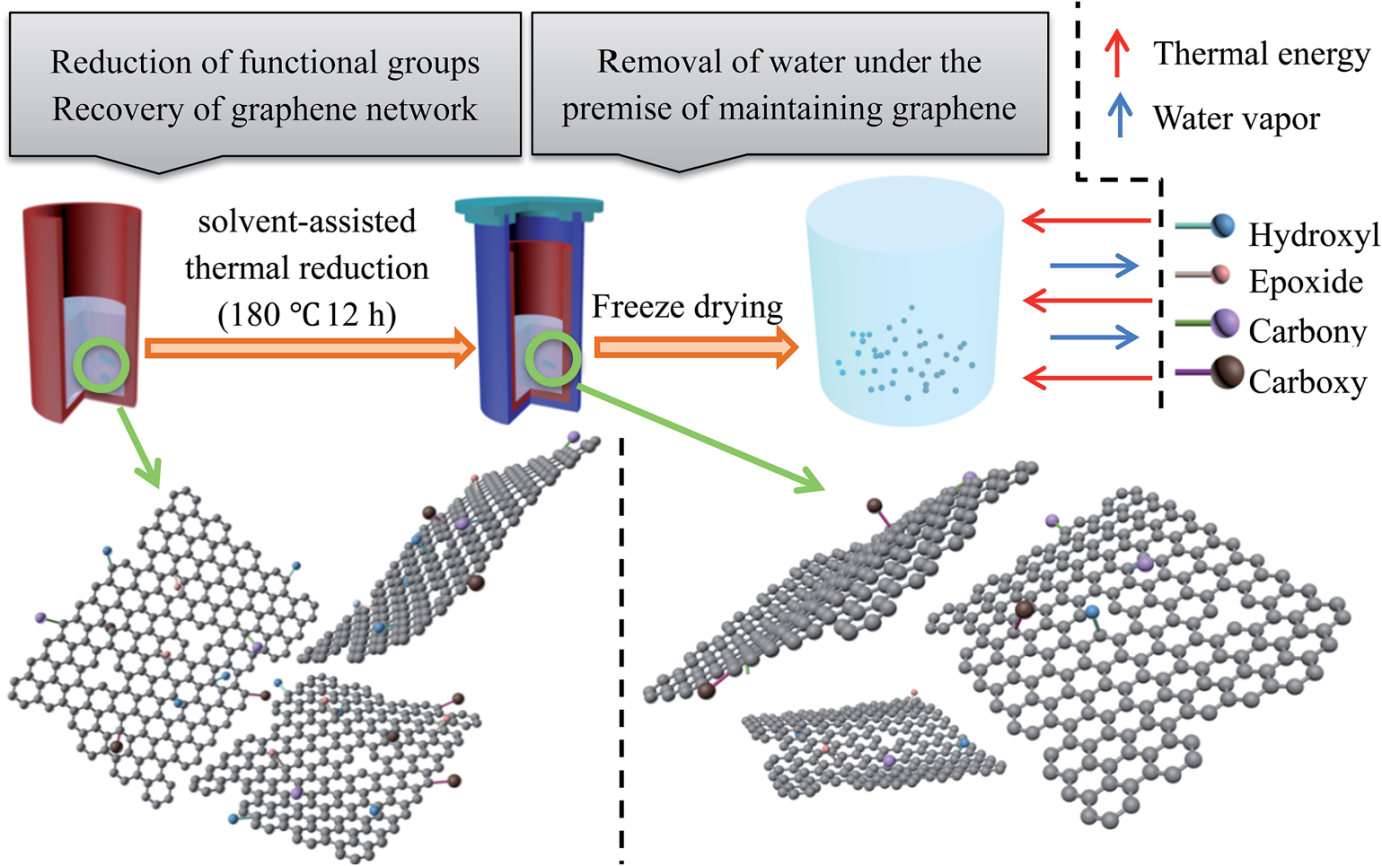
Fabrication process for rGO-M.

NMGM was chosen as the raw material to utilize its characteristics that are different from flake graphite. NMGM, a kind of abundant graphite mineral resource, has great potential for research and deep processing. As a common mineral resource, the carbon content of flake graphite mineral is very low, but flake graphite can be produced by a conventional floatation separation process because of the large diameter of the graphite. In contrast, although the grade of flake graphite mineral is higher than NMGM, it is more difficult to purify NMGM because its impurities are wrapped in ores due to the small diameter of graphite. This novel application of microcrystalline graphite, where it is used to prepare graphene which is then applied to microwave-absorbing materials, is considered to be able to effectively improve the processing technology and accessional value of microcrystalline mineral resources and broaden its application field. The illustration for the fabrication process is shown in Fig. 1.

### 3.1 Morphology and crystalline phase

**Fig. 2:**
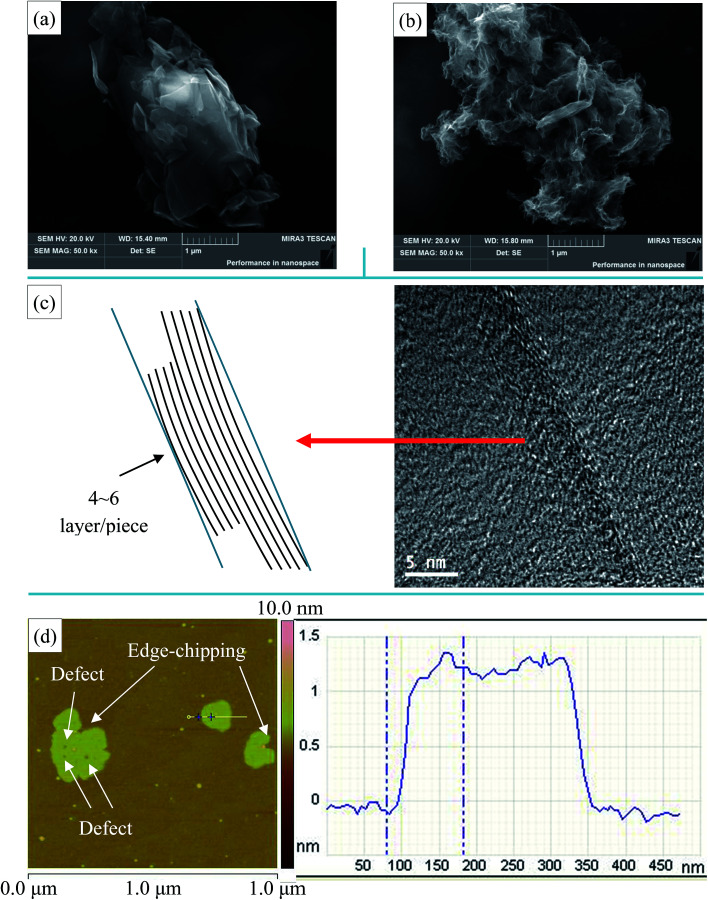
(a) SEM image of NMGM, (b) SEM image of rGO-M, (c) TEM image of rGO-M, and (d) AFM image of rGO-M.

The morphologies of NMGM and reduced graphene oxide prepared from natural microcrystalline graphite (rGO-M) were characterized by SEM, TEM and AFM (Fig. 2). It was found that there is an obvious difference between the morphology of NMGM and that of flake graphite minerals (FGM), and the lamellae of NMGM are very fragmented when compared to those of FGM. In Fig. 2(a), NMGM is a granular material stacked with graphite flakes of different sizes. The smaller graphite sheets, whose size is between 0.2 and 1 microns, are irregularly attached to the larger sheets, while the dimension in the length direction of the larger sheets is over 5 microns, and the width is about 2 microns. Fig. 2(b–d) show the microstructure of the rGO-M through different presentations. In Fig. 2(b), when the SEM photo is enlarged to 50.0k×, the picture shows a flexible structure similar to silk, which gives a certain degree of bending. The particle is stacked with graphene layers with various dimensions which match the structure of NMGM. Because of the destruction in the process of oxidation, the edges of the particles have been damaged and some defects have appeared inside the layers. The TEM image (Fig. 2(c)) reveals the large-small layer stacking structure from a smaller scale. It can be seen clearly that a piece of graphene with a diameter of around 10 nm is attached to a much larger sheet of graphene, and by adding a reference line, it can be found that the graphene layer has a tiny degree of bending. The thickness of each graphene piece is between 4 and 6 layers. According to the AFM image of rGO-M (Fig. 2(d)), the transverse dimension of the graphene lamellae is about 0.7 μm and the thickness is about 1.3 nm. Both the edges and the interior of the lamellae can be seen with defects due to spalling of the lamellae, which are consistent with those observed in the SEM image.

**Fig. 3:**
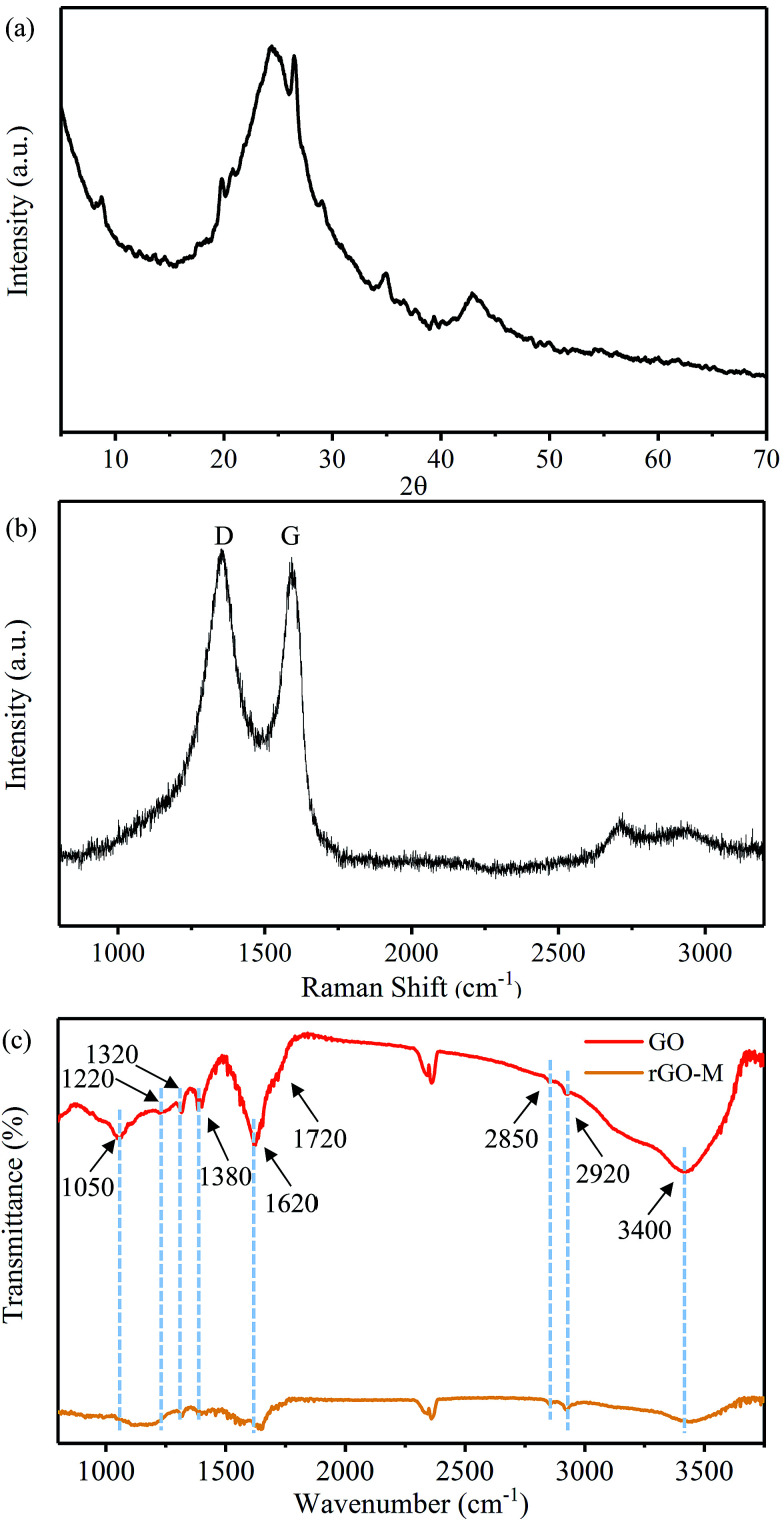
Crystal structure of rGO-M: (a) XRD, (b) Raman, and (c) FT-IR.

Fig. 3(a) shows the XRD pattern of the rGO-M. It is difficult to find a clear diffraction peak in the image, while only a wide peak can be seen at 2*θ* = 20–30°, and a relatively obvious graphite diffraction peak (100) appears at 2*θ* = 42.5°. This indicates that through the above experimental treatment, the graphene sheet was damaged and piled irregularly, and retained a portion of the graphite structure. The Raman spectrum (Fig. 3(b)) of the rGO-M shows the characteristic D and G bands of carbon at around 1360 cm^−1^ and 1580 cm^−1^. The value of *I*_D_/*I*_G_ is 1.42, and furthermore, the average crystallite size of the defect-free domains within the graphene is 23.788 nm.^39–41^ This shows that, compared with the perfect graphite crystal, the number of defects in the sample has been increased, and the graphite lamellae tend to be disordered, which is caused by the oxidation–reduction process. At the same time, the strong G peak also indicates that a large amount of carbon in the sample exists in the sp^2^ hybridized form and retains the carbon ring structure. Fig. 3(c) shows the FT-IR spectra of GO and rGO-M, and the peak positions and their assignments can be easily obtained and are described in Table 1. A summary of the information in Fig. 3(c) and Table 1 is as follows: the peaks of GO centered at 1620, 1720, 1050, 1220 (1320) and 3400 cm^−1^ are the stretching vibration peaks produced by the C=C, C=O, C–O–C, C–OH and O–H functionalities, while the peak at 1380 cm^−1^ represents the bending vibration of C–H.^42–44^ After reduction, the characteristic peaks of all oxygen-containing groups are weakened or even tend to disappear, which indicates that a number of oxygen-containing groups have been removed.

**Table 1:** FT-IR spectra of GO and rGO-M

Assignment	Stretching vibration bands						Bending vibration bands
	C–H	C=C	C=O	C–O–C	C–OH(COOH)	O–H	C–H
GO	2850, 2920	1620	1720	1050	1220, 1320	3400	1380
rGO-M	2850, 2920	1630	—	1060	1220, 1320	3420	1380

**Fig. 4:**
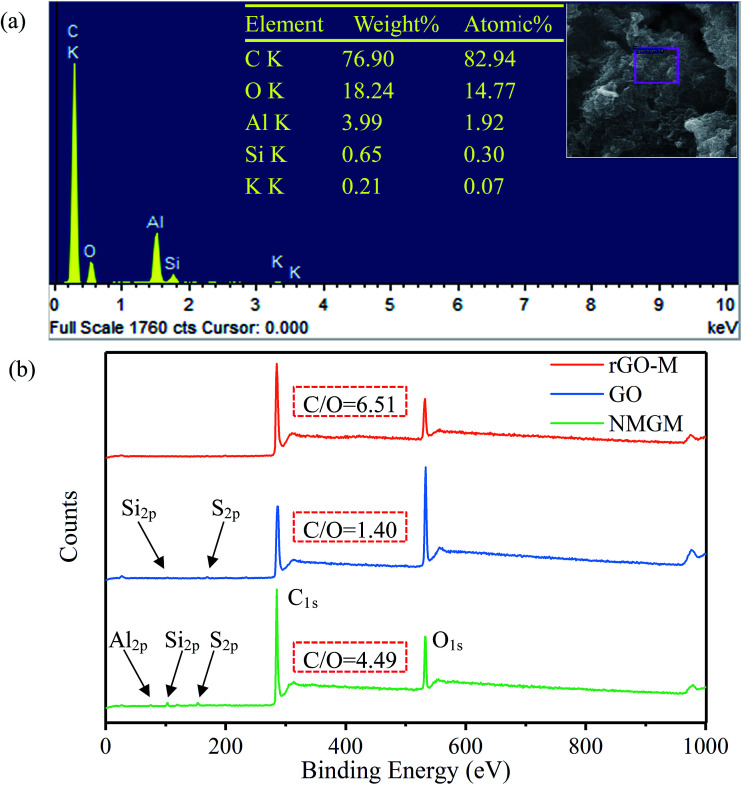
Analysis of element composition: (a) EDS of rGO-M and (b) XPS of preparation process.

The element changes in the oxidation–reduction process were investigated as well. In order to further study the elements of rGO-M, EDS of the sample was carried out. It can be found from Fig. 4(a) that the molar ratio of carbon and oxygen is 5.6, indicating a better reduction process, but there are still some oxygen-containing functional groups. Because the aluminum plate was used as the carrier during the test, and the sample was too thin, the aluminum element was detected. In Fig. 4(b), the element changes in the experimental process were characterized through full XPS. The NMGM spectrum indicates the existence of impurities of Al, Si and S. Compared with the original ore, the peak intensity of Al_2p_, Si_2p_ and S_2p_ gradually decreases and finally disappears during the preparation process of rGO-M from NMGN, indicating that impurities are greatly removed during liquid phase processing. Compared with the GO and rGO-M spectra, a significant change in the ratio of the C peak to the O peak shows that the reduction of the sample is successful. The effects of oxidation and reduction can be quantitatively characterized by the atomic ratio of carbon to oxygen (Table 2). The C/O ratio changed from 4.49 for NMGN to 1.40 for GO, a definite demonstration that a large number of oxygen-containing functional groups were grafted onto the carbon atom layer. After reduction, the C/O ratio increased to 6.5 for rGO-M, which indicated that GO has been largely reduced.

**Table 2:** The molar ratio of C and O, the chemical states of C elements and their molar ratios in samples

Samples	C (at%)	O (at%)	C/O	C–C/C=C (at%)	C–O (at%)	C=O(O–C=O) (at%)	C–O–C (at%)
NMGM	77.94	17.36	4.49	93.99	—	6.01	—
GO	57.44	40.93	1.40	47.65	42.66	9.69	—
rGO-M	82.40	12.65	6.51	55.58	19.52	—	24.90

**Fig. 5:**
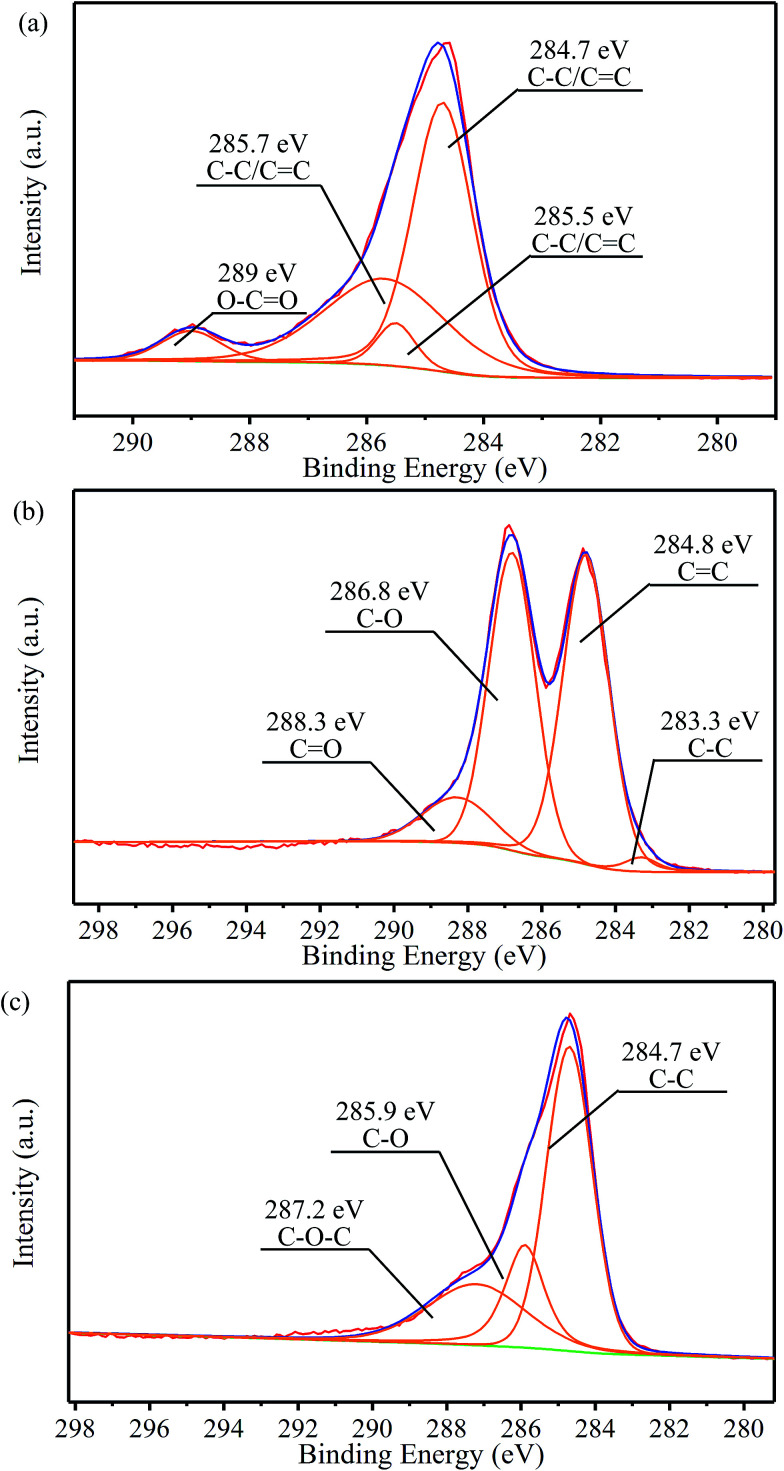
C_1s_ XPS spectra of (a) NMGM, (b) GO and (c) rGO-M.

In order to quantitatively analyze the change of functional groups and components in the preparation process, peak fitting of NMGM, GO, and rGO-M by C_1s_ was carried out and the results are as follows. The high-resolution C_1s_ spectra of the sample were deconvoluted into some different peaks that correspond to carbon atoms in different binding states (Fig. 5). For NMGM, the peaks centered at 284.7, 285.5 and 285.7 eV can be taken as C–C/C=C functional groups, and 289 eV as O–C=O functional groups (Fig. 5(a)). The data above show that the graphite ring structure is intact and that only a small amount of the carboxyl exists. In Fig. 5(b), GO possesses many heterocarbon components, such as the functional groups of C–C (283.3 eV), C=C (284.8 eV), C–O (286.8 eV), and C=O (288.3 eV). The results above suggest that GO contains various oxygen-containing functional groups. Through the previous characterization, the reduction process can obviously remove these functional groups. A significant decrease in heterocarbon components is observed in the C_1s_ spectra in Fig. 5(c), confirming the success of the reduction process again. For rGO-M, the peaks centered at 284.7 eV, 285.9 eV and 287.2 eV can be attributed to the C–C, C–O, and C–O–C functional groups, respectively. Table 2 shows the chemical states of the C elements and their molar ratios in the samples.^45–49^

### 3.2 Electromagnetic parameters and reflection loss

**Fig. 6:**
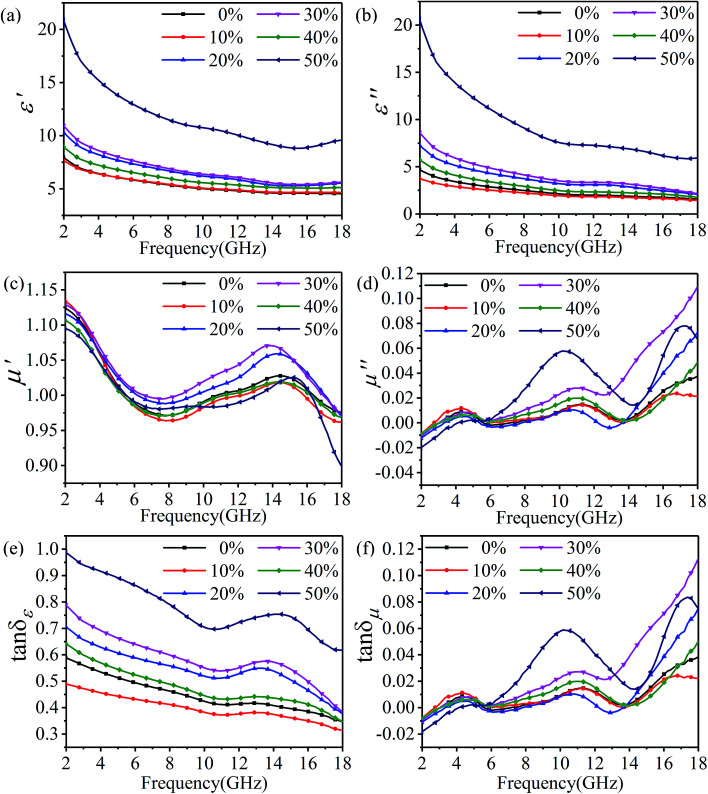
Electromagnetic parameters of rGO-M/paraffin in different ethanol concentrations: real (a) and imaginary (b) parts of permittivity, real (c) and imaginary (d) parts of permeability, dielectric loss tangent (e) and magnetic loss tangent (f).

In order to research the effects of rGO-M as an absorbent, ethanol concentration was used as a variable to explore the microwave absorbing performance. Fig. 6(a and b) illustrates the real (*ε*′) and imaginary (*ε*′′) parts of the relative complex permittivity from 2 to 18 GHz. As a whole, both the real part and the imaginary part present a downward trend across the whole band. Except for the samples with 50% ethanol concentration, the range of which is more than 10, the trend of the remaining samples varies very slowly, and the range of variation remains within 7 (*ε*′ from 3.03 to 5.56 and *ε*′′ from 2.30 to 6.49). It can be seen in Fig. 6(c and d) that, in all samples, either the real (*μ*′) or imaginary (*μ*′′) parts of the relative complex permeability show a certain trend but the change range is very small (<0.25), and this value is consistent with the paraffin matrix material, so there is almost no value for analysis. The result of the relative complex permeability indicates that the material is almost non-magnetic. The change of the dielectric loss tangent (tan *δ*_*ε*_) with frequency in different ethanol concentrations is shown in Fig. 6(e). With the increase in the frequency, the change in the trend of tan *δ*_*ε*_ in different ethanol concentrations is to decrease at first, then increase, and finally return to decreasing, but the change is not significant, and the variation is between 0.3 and 1. In Fig. 6(f), as the frequency increases, the magnetoelectric loss tangent (tan *δ*_*μ*_) shows the opposite trend with tan *δ*_*ε*_, that is, increasing in the range of −0.02 to 0.12. Subsequently, referring to the data in front of *μ*′ and *μ*′′, it can be concluded that the rGO-M is not an absorber for magnetic loss.

**Fig. 7:**
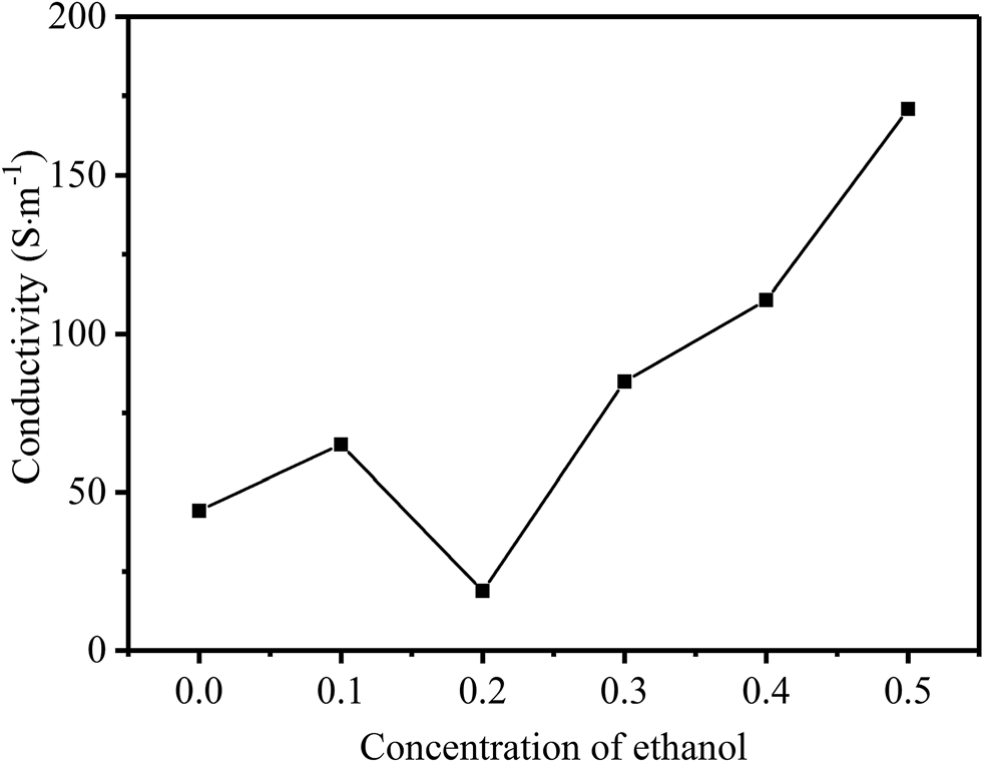
Line chart of conductivity with different ethanol concentrations.

According to the discussion above, it can be confirmed that rGO-M is a kind of electromagnetic wave absorber with electric loss. In order to subsequently study its electrical properties, the electrical conductivity changes with the concentration of ethanol were tested (Fig. 7). With the increase in the concentration of ethanol, the overall trend of rGO-M conductivity is to increase. When the ethanol is not added, the conductivity is only a few tens of S m^−1^. As the concentration of ethanol increases, the conductivity can reach hundreds of S m^−1^, which shows a difference in orders of magnitude. Under high temperature and high pressure conditions, with the removal of oxygen-containing functional groups, the self-assembly process of graphene sheets will occur in the presence of noncovalent interactions, which affects the conductivity of the graphene prepared. The increase in the ethanol concentration makes the degree of self-assembly increase, resulting in the reduction of isolated graphene sheets. The conductivity increases with the increase in free electrons in the structure.

Based on the generalized transmission line theory and the normalized input impedance *Z*_in_ of a metal-backed electromagnetic absorbing layer, reflection losses of the rGO-M/paraffin composites can be calculated from the measured dielectric characteristics according to the following equations (*d* = 4 mm).^21^1
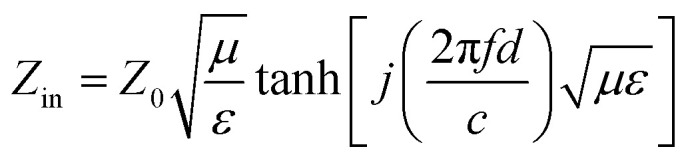
2
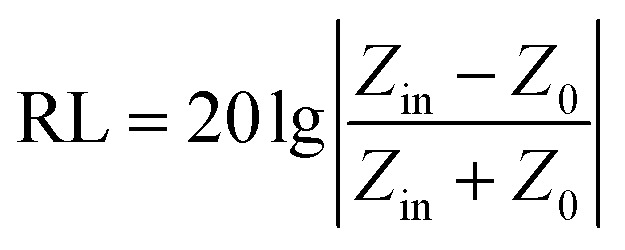


**Fig. 8:**
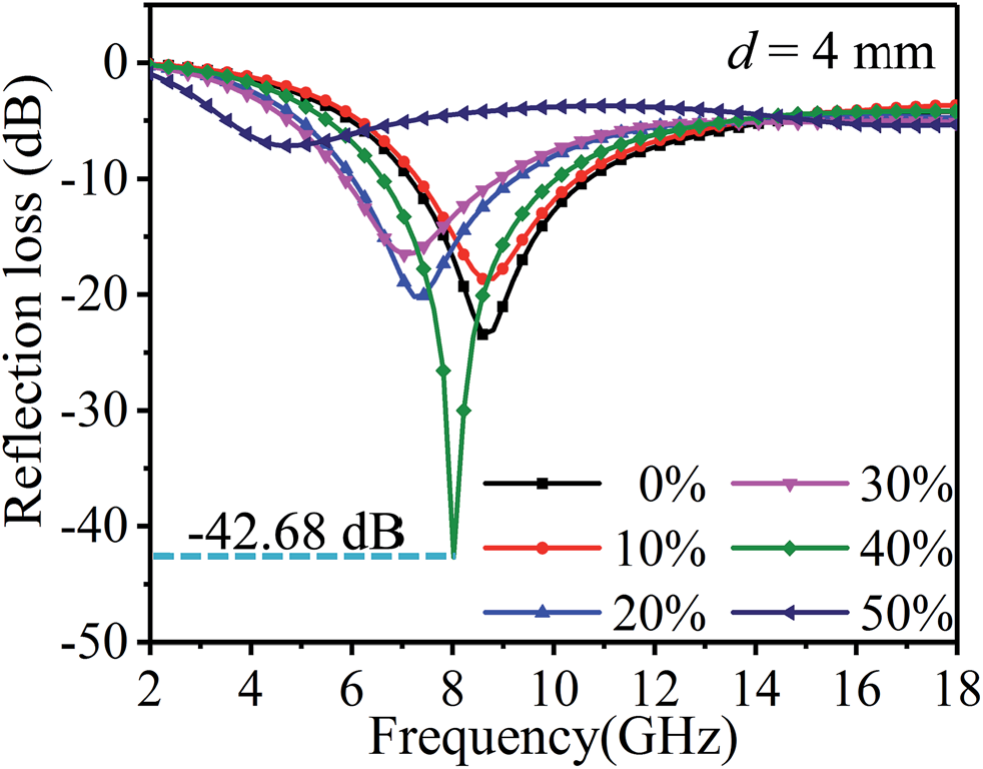
Reflection loss of rGO-M/paraffin in different ethanol concentrations.

Fig. 8 shows the influence of ethanol concentration and different frequencies on the reflection loss and Table 3 is the comparison of the wave absorbing performance. As can be seen from the figure and table, the main absorption peak of the sample is mainly concentrated between 6–10 GHz. With the increase in the ethanol concentration, the reflection loss of samples shows a more complex trend. In addition to the samples with ethanol concentrations of 40% and 50%, with the increase in the ethanol concentration, the effective absorption band shifts to low frequency and the wave absorption performance is optimized. At the ethanol concentration of 40%, the maximum absolute value of the optimum reflection loss can be obtained, and the value is −42.68 dB, at 8.02 GHz. By contrast, at the ethanol concentration of 50%, the minimum absolute value of the optimum reflection loss can be obtained at 16.99 GHz, with the value of −5.43 dB. Based on −5 dB, the sample with the highest absorption bandwidth is the sample with 30% ethanol concentration, and the bandwidth value is 11.70 GHz. When −10 dB is considered as a benchmark, the sample with the highest bandwidth is the sample with 40% ethanol concentration, and the value is 3.32 GHz.

**Table 3:** Reflection loss of the rGO-M/paraffin in different ethanol concentrations (*d* = 4 mm)

Ethanol concentration	Max. reflection loss (dB)	*f*_m_ (GHz)	Reflection loss <−5 dB		Reflection loss <−10 dB	
			Frequency range (GHz)	Bandwidth (GHz)	Frequency range (GHz)	Bandwidth (GHz)
0%	−23.44	8.6	6.07–14.45	8.38	7.24–10.55	3.31
10%	−18.66	8.6	6.26–13.87	7.61	7.43–10.36	2.93
20%	−20.17	7.24	5.09–13.87	8.78	6.07–9.19	3.12
30%	−16.53	7.04	4.90–16.60	11.70	5.88–8.80	2.92
40%	−42.68	8.02	5.68–13.48	7.80	6.65–9.97	3.32
50%	−5.43	16.99	3.53–7.04	6.28	—	—
			15.23–18.00			

**Fig. 9:**
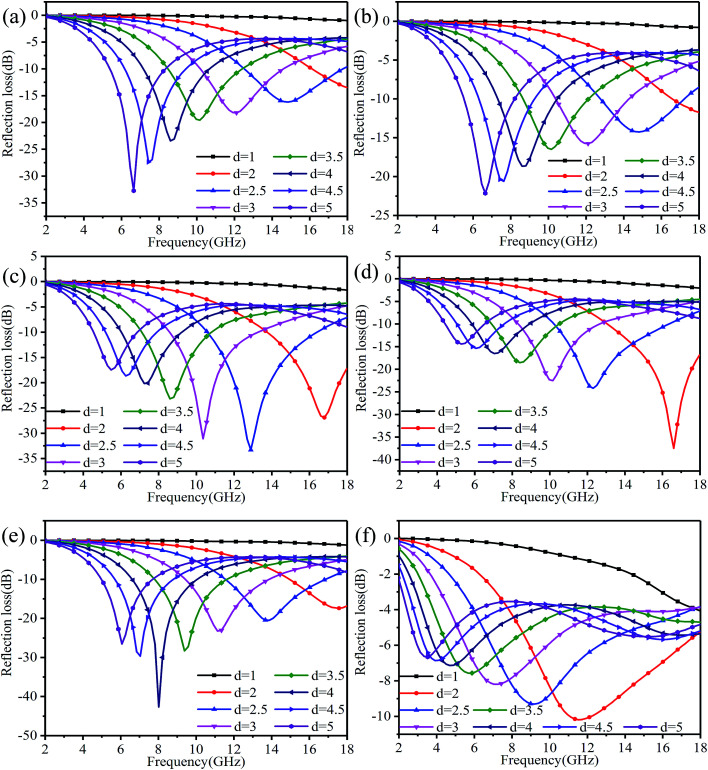
Reflection loss of rGO-M/paraffin under different thicknesses: (a) to (f) – ethanol concentrations 0%, 10%, 20%, 30%, 40%, and 50%.

According to the theory of linear transmission, the thickness of the material has an influence on its electromagnetic absorption performance. The reflection loss of rGO-M/paraffin under different thickness conditions is show in Fig. 9, and is used to investigate the change trend when the thickness is changed. As a whole, when the thickness is greater than 1 mm, as the thickness increases, the effective absorption bandwidth of the materials moves to bands with low frequencies, with an obvious law of motion. At 0% and 10% ethanol concentrations, the effective absorption bandwidth is narrow and the absorption intensity increases, while for 20% and 30% ethanol concentrations, it is the opposite. In Fig. 9(e), as the thickness increases, the peak value of the absorption intensity is not obvious, and the sudden enhancement only occurs when *d* = 4 mm. When the ethanol concentration is 50%, the change in the effective absorption bandwidth is very obvious, but the difference in the absorption peak intensity is not big, and no special point appears.

**Fig. 10:**
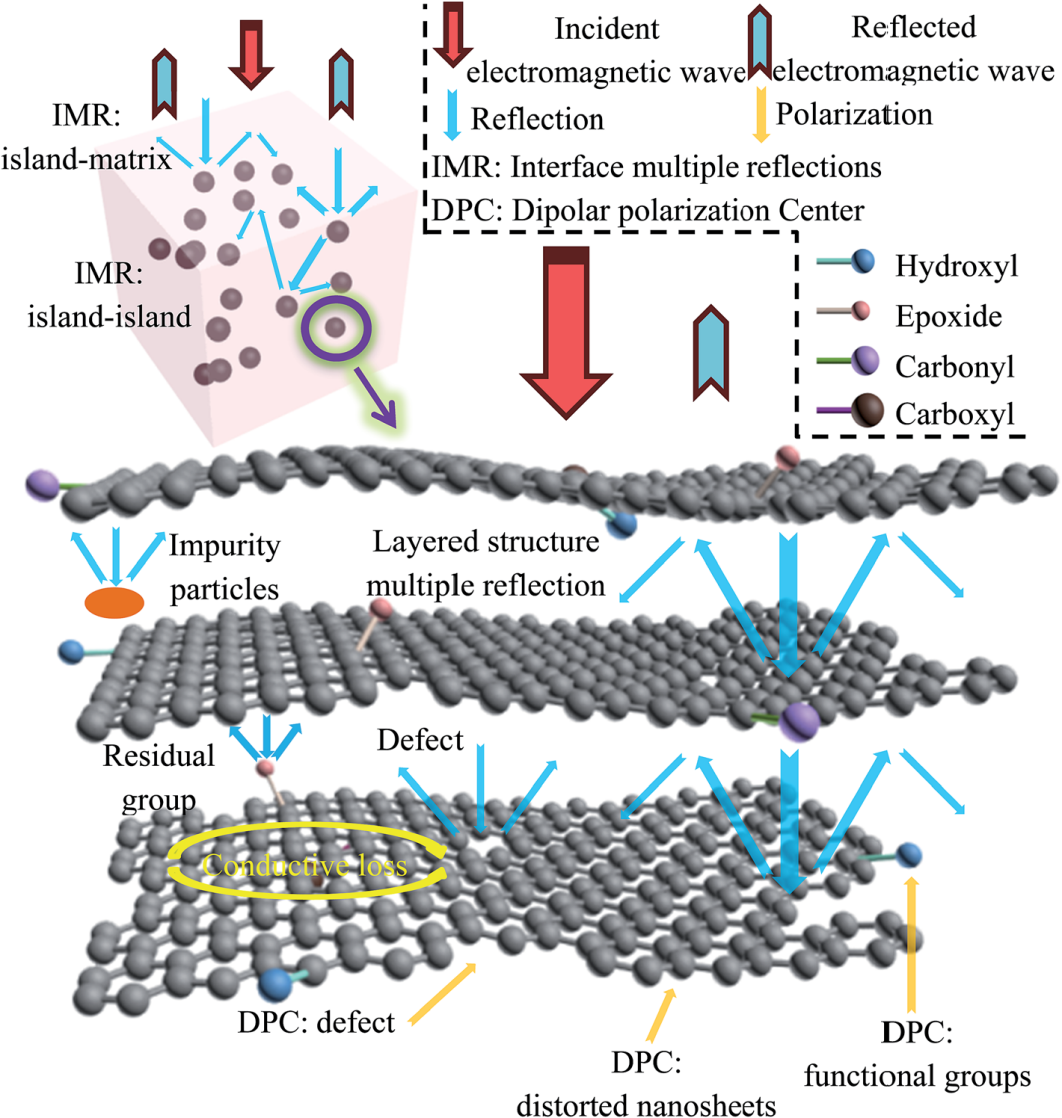
Schematic illustration of possible electromagnetic wave absorption mechanisms.

The oxidation process can fully disperse the graphite ore, and release the impurities wrapped in the ore into the solution. Solvent-assisted thermal reduction can promote the self-assembly of graphene sheets, and the impure particles are prevented from being wrapped again. What is more, the electronic structure of graphene is restored, giving the material a moderate conductivity, and the residual oxygen-containing functional groups can help to reduce the conductivity of the material, thus improving the impedance matching performance. Fig. 10 shows the possible electromagnetic wave absorption mechanisms of rGO-M/paraffin. The superiority of rGO-M/paraffin can be attributed to many features. Natural microcrystalline graphite is a small crystal mineral with many impurities. Most of these impurities exist in the form of inclusions, which are difficult to completely remove in the preparation process. In addition, the reduction process does not completely remove oxygen-containing functional groups from the graphene sheets. The presence of impurities and oxygen-containing functional groups can improve the impedance matching performance of rGO-M/paraffin and provide the prerequisite for absorbing electromagnetic waves.^21^ In rGO-M/paraffin, rGO-M and impurities can be regarded as isolated islands in the paraffin matrix, and form a large number of interfaces between the islands and the matrix. Reflection and refraction occur when the electromagnetic wave meets the interface between the islands and the matrix in the transmission process. The reflected electromagnetic wave continues to propagate in the substrate until other interfaces are encountered, by which point the wave may have reflected many times inside the material. Therefore, the existence of these isolated islands greatly increases the transmission path of electromagnetic waves in the material and such propagation would cause electromagnetic waves to lose some of their energy. The reflection loss of energy not only occurs among large numbers of isolated islands, but also within the isolated islands. rGO-M has a two-dimensional lamellar structure, causing multiple reflection losses. As for rGO-M, there are residual defects and groups on graphene sheets, corresponding to the formation of many micro-interfaces, which mean the electromagnetic wave needs to consume more energy to continue to spread. The two structural characteristics further strengthen the reflection loss. In conclusion, the electrical properties of graphene’s isolated islands are indeed conducive to absorbing electromagnetic waves. The dielectric property results suggest that the rGO-M has a strong dielectric loss capability, which can effectively dissipate electrical energy. In the composite, the rGO-M forms many conductive micro-networks, and if an external electric field is applied to the material, the energy will be induced into many microcurrents, generating a strong conductive loss, and resulting in the increase in the electromagnetic wave absorption capability. As mentioned above, according to dielectric spectroscopy, there are many interfaces in the materials, where polarization loss may occur. When the materials were exposed to an electromagnetic field, a small amount of unneutralized positive and negative free charges accumulated on various interfaces and abundant boundaries, and then plenty of dipoles were formed once these charges were trapped by defects. From the discussion above, it can be concluded that the enhanced microwave absorption performance of rGO-M/paraffin is ascribed to the better impedance match, the special hierarchically porous system and its polycrystalline structure.^50–53^

## 4. Conclusions

In summary, microcrystalline graphene oxide was successfully fabricated by a solvent-assisted thermal reduction. The recovery of conductive properties can achieve the desired effect with lower mass fraction conditions. The as-received microcrystalline graphene oxide has a unique conjugated graphene network and amorphous nanostructure which is usually required for applications in microwave absorption. With adjustable electrical resistivity, it is rather easy for the as-produced microcrystalline graphene oxide to be transferred into well distributed bulk materials. Based on the conjugated graphene network and amorphous nanostructure, microcrystalline graphene oxide demonstrates an extremely superior microwave absorption performance in the GHz band. The graphene/paraffin composites with different thicknesses show different trends and can be regulated according to requirements. Meanwhile, the effective bandwidths below −5 dB and −10 dB are 11.7 GHz and 3.32 GHz respectively, and the maximum reflection loss can reach up to −42.68 dB. rGO-M/paraffin composites made by solvent-assisted thermal reduction as electromagnetic wave absorbers can significantly improve absorption performance in the low frequency band.

## Conflicts of interest

There are no conflicts to declare.

## Supplementary Material
